# The Role of Bifidobacteria in Predictive and Preventive Medicine: A Focus on Eczema and Hypercholesterolemia

**DOI:** 10.3390/microorganisms9040836

**Published:** 2021-04-14

**Authors:** Luisa Marras, Michele Caputo, Sonia Bisicchia, Matteo Soato, Giacomo Bertolino, Susanna Vaccaro, Rosanna Inturri

**Affiliations:** 1 Department of Medical Science and Public Health, University of Cagliari, 09042 Cagliari, Italy; luisa.marras@unica.it; 2 Fidia Farmaceutici S.p.A., R&D Unità locale Fidia Research sud, Contrada Pizzuta, 96017 Noto, Italy; mcaputo@fidiapharma.it (M.C.); sbisicchia@fidiapharma.it (S.B.); svaccaro@fidiapharma.it (S.V.); 3 Fidia Farmaceutici S.p.A., R&D Abano Terme, 35031 Abano Terme, Italy; msoato@fidiapharma.it; 4 Pharmaceutical Department, Azienda Ospedaliero Universitaria di Cagliari, 09042 Cagliari, Italy; giacomo.bertolino1985@gmail.com

**Keywords:** *Bidifobacterium longum*, *Bidifobacterium bifidum*, dermatitis syndrome, eczema, cholesterol, SCFAs, BDNF, gut microbiota, skin microbiota, COVID-19

## Abstract

Bifidobacteria colonize the human gastrointestinal tract early on in life, their interaction with the host starting soon after birth. The health benefits are strain specific and could be due to the produced polysaccharides. The consumption of probiotics may prevent obesity, irritable bowel syndrome, eczema or atopic dermatitis, and asthma. Non-replicative strains of *Bifidobacterium longum* (NCC3001 and NCC2705) promote the differentiation of normal human epidermal keratinocytes (NHEKs), inducing a high expression of differentiation markers (keratin —KRT1—, and transglutaminase —TGM1—) and pro-regeneration markers (cathepsins), including β-defensin-1, which plays an important role in modulating the cutaneous immune response. Strains belonging to the genera *Bifidobacterium* and *Lactobacillus* can increase tight-junction proteins in NHEKs and enhance barrier function. Bifidobacteria and lactobacilli may be used as prophylactic or therapeutic agents towards enteric pathogens, antibiotic-associated diarrhea, lactose intolerance, ulcerative colitis, irritable bowel syndrome, colorectal cancer, cholesterol reduction, and control of obesity and metabolic disorders. *Bifidobacterium bifidum* showed an in vitro capability of lowering cholesterol levels thanks to its absorption into the bacterial membrane. Several strains of the species *Lactobacillus acidophilus*, *L. delbrueckii* subsp. *bulgaricus*, *L. casei*, and *L. gasseri* led to a reduced amount of serum cholesterol due to their ability to assimilate cholesterol (in vitro). *Lactococcus lactis* KF147 and *Lactobacillus plantarum* Lp81 have also been shown to reduce cholesterol levels by 12%. Clarifying the specific health mechanisms of *Bifidobacterium* and *Lactobacillus* strains in preventing high-cost pathologies could be useful for delineating effective guidelines for the treatment of infants and adults.

## 1. Introduction

The human body is made up of many kinds of cells: skin cells, muscle cells, neurons, and blood cells. These different kinds of cells develop and function under the guidance of a single set of genes, shared by all human cells called the *human genome* [1].

In addition, the entire surface of the human body (skin, lining of the nasal passages, lungs, digestive, and urogenital tracts) contains about 100 trillion bacteria cells, viruses, bacteriophages, and other microorganisms belonging to the *Archaea*, *Bacteria*, and *Eukarya* domains. These microorganisms have their own gene set and mainly belong to non-pathogenic species or, more rarely, to pathogenic species that have co-evolved with the host. Nowadays, the term *human microbiota* is already widely used to indicate the host-associated microbial communities (1300 g and 1.5 L) [2,3] and, likewise, *human microbiome* indicates the entire collection of genes belonging to microorganisms which colonize the human body. Numerically speaking, the microbial cells that colonize the human body make up three quarters of the host’s total cells. The genome of the microbiota (microbiome) contains a number of genes that is 400 times higher than the total number of human genes (~20,000) [1,2]. These numbers suggest that the microbiome plays an important evolutionary role in human health, exerting specific genetic and metabolic functions that have not evolved in the human host [2,3,4,5,6,7]. 

Microbial colonization is a very complex process that begins immediately after birth. Babies born through the vaginal canal come into contact with microorganisms coming from the mother’s genitourinary tract; the next source of microbes is breast milk. The microbiota interacts with human cells through specific markers present on the cells [5]. During the first three years of life, a child comes into contact with environmental microbes from food, soil, plants and pets, and this is the start of individual microbiome selection. Most environmental microbes are unable to live in the human body and thus will not become stable and resilient in the individual’s lifespan. Although there is genetic diversity in the microbiome, due to adaptation between microorganisms and the host and also the influence of different lifestyles and various physiological and pathophysiological factors, the vast majority of humans all share the same microbial communities in a given habitat. The set of microbes that constitutes the *core* of the mature human microbiome is the result of a mutual selection process. The *core* of a human microbiome is constituted by a set of genes present in the whole or a specific part of the human body and is characteristic for each microbiome. The microbiome is also made up of a variable part that is represented by a set of genes that differs from person to person depending on genotype, physiological status, immune system, pathobiology, lifestyle (including diet), and environment. Although the microbiota is quite stable over time, new genes can be included, and others excluded [5].

The main microbial communities are those in the gut, mouth, and skin (pores, sweat glands, and hair shafts) and differ in density, characteristics, and functional role [5]. The microbiome composition has a complex biogeography, varying from place to place, depending on environmental conditions, for example, i.e., oxygen concentration, moist or oily areas like the armpits or nasal creases have higher and different microbes compared to dry areas like the arms and legs. Many differences in human microbiomes are observed when they are classified on the basis of the bacterial species. When the microbiomes are classified on the basis of functional capabilities, such as the ability to digest carbohydrates, synthesize vitamins, or destroy toxins, they are quite similar [8].

The human mouth hosts about 100–200 species located in many different micro-habitats, including tooth surfaces, tongue, cheeks, and gums [1]. 

The human gut microbiome contains the largest, densest, and most diverse microbial community (up to 100 billion to one trillion cells per milliliter in the large intestine) [1]. This acts as a highly efficient bioreactor, helping to extract energy and nutrients from the food we eat, producing short-chain fatty acids (SCFAs) and vitamins; driving functional roles for the host, such as maturation and modulation of the immune system, intestinal epithelial maturation, and regulation of mucosal physiology; and protecting against pathogens, such as *Clostridium difficile* [9,10,11,12,13]. 

The human skin microbiome hosts strains belonging to the species *Staphylococcus epidermidis*, which are able to keep away pathogens, such as *Staphylococcus aureus*, producing compounds that inhibit their growth [1,3,14,15,16,17]. 

The microbiome balance (homeostasis) is called eubiosis and is characterized by a preponderance of potentially beneficial species. The aberrant microbiome deviations from homeostasis are called dysbiosis, which is a condition related to several diseases [18]. 

Advances in high-throughput sequencing, bioinformatics, systems biology, computational biology, and the enrichment of the ‘-omics’ database have contributed to a better understanding of many human physiological and pathological conditions and the microbiome role in the human body, helping to design multiparameter systematic strategies for each human individual condition. This new knowledge could lead to the development of individualized diagnostics, patient-specific therapy, therapy approaches using autologous cells, and thus to the individual care of patients. The main phases in prognostic, diagnostic, preventive, and/or therapeutic research nowadays are delineated and start from healthy people (phase 1) to reaching patients affected by specific diseases (phases 2, 3, and 4), performing randomized clinical trials and observational studies. From this kind of studies, is it possible to generate a prognostic index that can be applied as a prognostic score in clinical practice [19]. The analysis of specific biomarkers in the human host and mechanisms through microbes and produced metabolites’ interaction with specific host receptor can offer new possible strategies in the prevention of many pathologies.

Immune disorders and low levels of SCFAs can be observed in pathologies seemingly distant from each other, such as cardiovascular diseases and skin disorders. Leukocytosis and in particular monocytosis is related to an increased risk of atherosclerosis in patients with high total plasmatic cholesterol levels; on the other hand, dendritic cell-mediated T-helper 2 (Th2) disorders are related to severe atopic dermatitis [20,21]. 

The European Association for Predictive, Preventive and Personalized Medicine underlines the importance of targeted prevention in nutrition, behavior, and physical activity metabolic for cardiovascular diseases and immunological disorders [19]. Increasing evidence indicates that the gut microbiota is strongly associated with a reduced risk of necrotizing enterocolitis in infants, with treatment of acute infection diarrhea and reduced risk of antibiotic-associated diarrhea and colon rectal cancer and lactose maldigestion in children and adults [18,22,23,24]. Moreover, the genera *Bifidobacterium* and *Lactobacillus* are associated with a decrease of symptoms of inflammatory bowel disease, vaginal infections, a balanced human metabolism, and exert a key role in type 1 diabetes (T1D) through the regulation of pro- and anti-inflammatory cytokine production [25]. Bifidobacteria and lactobacilli have also shown interesting properties in reducing cholesterol levels in vitro and in vivo [26,27,28,29,30,31,32]. Considering the statin side-effects, an advanced knowledge on the effects of bifidobacterial-produced metabolites could be useful in the research of new alternative therapies [33]. 

The enrichment of the ‘-omics’ database has contributed to a better understanding of the microbial role in drug metabolism, leading to the concept of ‘pharmacomicrobiomics’. The microbiota take part in drug metabolism (methotrexate, gemcitabine) and activate metabolites through different kinds of chemical reactions, such as reduction, acetylation, hydrolysis, dehydroxylation, dealkylation, decarboxylation, deamination, and deconjugation. Thus, specific classes of chemotherapeutic agents can be influenced both in their efficacy and toxicity by chemical signaling cascades activated by gut microbiota [34]. Some strains in the gut microbiota have a specific metabolic pathway, which includes the enzyme β-glucoronidase that cleaves the glucuronide portion from the inactive metabolite of irinotecan, releasing its active metabolite and causing diarrhea [34]. The mouse fibrosarcoma MCA205 progression has been shown to be controlled by cytotoxic T-lymphocyte-associated protein 4 (CTLA-4) antibodies in pathogen-free mouse but not in germ-free mouse. Gut microbes belonging to the genus *Bifidobacterium* exhibit an antitumor activity in patients treated with the Programmed Death-Ligand 1 (PD-L1) monoclonal antibody, through the induction and increase in T cells [34]. 

The knowledge about gut microbiota has paved the way to a better understanding of skin microbiota [35,36,37,38].

In vitro and in vivo studies have examined the mechanisms behind many inflammatory skin conditions, such as those observed in atopic eczema and dermatitis [39,40]. Moreover, systematic reviews have given an overview on the advances in the understanding of gut microbiota effects, and clinical trials have been well organized to assess the efficacy in using strains belonging to the genus *Bifidobacterium* or *Lactobacillus* for treating eczema and other immunity-related disorders, such as metabolic syndrome, cardiovascular diseases, and diabetes mellitus type 2 (T2D) [25,33,41]. 

Hypercholesterolemia and eczema are two pathological conditions studied in personalized medicine and are linked by immune regulation, barrier permeability, and metabolites production by healthy strains. In particular, the beneficial role exerted by strains belonging to these genera or their metabolites seems to be related to their capability to stimulate the production of interleukin-10 (IL-10) and tumor necrosis factor (TGF-beta) by T cells and to control the T-helper 1 (Th1) and 2 (Th2) response. High levels of Th2 cytokines makes the skin permissive to *Staphylococcus aureus* colonization. Furthermore, bile acid deconjugation has a key role in the reduction of plasma and liver triglycerides and in the increased expression of human half-transporters belonging to G subfamily of adenosine triphosphate-binding cassette (ABC) transporters called ABCG5 and ABCG8 [33,42].

Therefore, such evidence has underlined the importance of developing strategies to restore and/or maintain the balance between species within each microbial community. A prebiotic strategy uses prebiotic substances (fructooligosaccharides —FOS— and galactooligosaccharides —GOS—) to favor the growth of beneficial microorganisms, a probiotic strategy uses safe strains that exert probiotic effects, while a symbiotic strategy uses probiotic and prebiotics together [43,44,45,46,47]. 

Projects, such as the human microbiome project and the project on European metagenomics of the human intestinal tract, have allowed characterization of the abundance, diversity, and functionality of genes present in all the microorganisms that live/do not live permanently in different sites of the human body; on the other hand, the knowledge about intracellular mechanisms and their specific link with gene expression has led to the definition of the genes involved and their biological roles in host health promotion [48,49,50]. Specific biomarkers could be considered to study host-specific phenotypes and to develop individualized therapies based on age, gender, and gene expression. Although some reviews of strains belonging to the genera *Bifidobacterium* and *Lactobacillus*, usually used as a probiotic component in food supplements, already exist [39,41], no recent data have been collected that focus the attention on bifidobacterial, from molecular research to host interaction.

The present review aims to clarify the specific health-promoting mechanisms of *Bifidobacterium* strains alone or in combination with lactobacilli in preventing eczema or dermatitis disorders and hypercholesterolemia in order to help delineate effective specific guidelines.

## 2. Gut Microbiota

Gut microbiota is constituted by a microbial community that behaves as a dynamic entity characterized by an equilibrium between stability and diversity. Gut colonization is a very complex process, and gestational age is a key factor in the establishment of gut microbiota. Preterm infants (<37 weeks) show an incomplete intestinal barrier and are exposed to hospitalization risk, which usually leads to antibiotic administration. For these infants, the colonization is more difficult, as their gut microbiota show a high level of opportunistic bacteria belonging to *Enterobacteriaceae* and *Enterococcus* instead of *Bifidobacterium* and *Bacteroides*. The functionality of gut microbiota is also compromised in preterm infants, and low levels of short-chain fatty acids (SCFAs) have been detected. Moreover, caesarean-delivered infants show reduced levels of various cytokines, and these immune disorders are associated with an increased risk of allergic and metabolic diseases [12]. During the first weeks of life, the microbiota of vaginally delivered and breastfed infants shows a prevalence of *Actinobacteria* and in particular the genus *Bifidobacterium*, with a dominance of the species *Bifidobacterium breve*, *Bifidobacterium bifidum*, and *Bifidobacterium longum* [51]. The intestine of these newborns is also colonized by *Lactobacillus* spp., with a predominance of *Lactobacillus rhamnosus* and *Lactobacillus gasseri.* The microbiota of caesarean-delivered and / or formula-fed infants is similar to the skin microbiota, with a prevalence of the genera *Staphylococcus*, *Corynebacterium*, and *Proprionibacterium* [52,53]. The bacterial migration from the maternal gastrointestinal tract to the mammary glands could happen through mononuclear immune cells via an endogenous cellular route [54]. Breast milk provides nutrients; human milk oligosaccharides, which selectively promote growth of beneficial microbes; and secretory immunoglobulins A (IgAs), which have a regulatory function for the immune system. The differences between the gut microbiota of breast- and formula-fed infants is also reflected in SCFA levels in the stool: propionate and butyrate display higher levels in formula-fed infant’s stool. The cessation of breastfeeding allows an important change in infants’ microbiota: a shift in the abundance of the genera *Bacteroides*, *Blautia*, and *Ruminococcus* and a decrease of *Bifidobacterium* and *Lactobacillus*. Other key factors in human gut composition are maternal diet, family lifestyle, geographical location, and host genotype. When the human gut microbiota is considered, it is an important process, including dispersal, local diversification, and environmental selection. The diversity in local microbial communities is the result of continuing selection in the bacterial pool that colonized the host. Local microbial diversification is the result of mutation and recombination processes between the strains, and in the context, a key role is that of phages, especially for microbes allocated in the same ecological niches [12].

There are few bacterial divisions in all the districts of the intestinal microbiota, also called enterotype, but in a constantly dynamic status [55]. These divisions are highly differentiated at the level of species and subspecies. Metagenomic studies have shown a predominance of five highly differentiated bacterial divisions, namely *Firmicutes*, *Bacteroidetes*, *Proteobacteria*, *Actinobacteria*, and *Fusobacteria*, whose distribution throughout the human gut is related to pH, bile salts, oxygen concentration, nutrients, water, and temperature [3,7,51]. The *Clostridium coccoides* group, which includes both cultivable and non-cultivable species, prevails in the *Firmicutes* phylum, with group diversity related to age and host health [56].

Many studies have been carried out to clarify the relationship between the microbiome, health, and disease [1,57,58].

Alterations in gut microbiota and mild inflammation can induce a chronic state of immune-mediated diseases. Antibiotic therapies and metabolic, cytotoxic, and pathogenetic stress can determine an altered relationship between *Firmicutes*/*Bacteroidetes* and a decrease in microbiota variability at the species level known as dysbiosis, which seems to exert an important role in metabolic, inflammatory, and allergic diseases [57,59,60,61]. Gut microbiota contributes to host metabolism through the fermentation of non-digestible polysaccharides of vegetable origin; the production of SCFAs, such as butyric, acetic, and propionic acid; and the production of vitamins (vitamin K and B12, riboflavin, thiamine, biotin, and folate). SCFAs are able to inhibit nuclear factor kappa B (NF-kB) and thus cytokine production, with effects on anti-inflammatory activity. On the contrary, the activation of toll-like receptors (TLRs) through the linking between gut bacteria and specific recognition sites on intestinal epithelial cells leads to activation of the immune system, resulting in a reduction of fat absorption and leptin levels. The balance between metabolic products and their own activity results in a healthy immune function of the gut epithelium [62]. The SCFA, mainly produced by *Bifidobacterium*, contribute to a reduction in cholesterol and to trophic properties due to the ability of butyrate to increase pro-differentiation and proangiogenic factors, such as brain-derived neurotrophic factor (BDNF), with a trophic effect on epithelial cells. BDNF is also involved in two-way communication between intestinal microbiota and the brain, in which the endocrine, immune, and nervous systems are also involved [63]. The structural function of gut microbiota includes the formation and stabilization of the gut epithelial barrier by strengthening the expression of cadherins for adherens junctions (AJs), claudins, occludin, and junctional adhesion molecules for tight junctions (TJs). The barrier effect of the gut microbiota has a protective role against pathogenic bacteria through the ability to recognize pathogen-associated molecular patterns (PAMPs), compete for nutrients and adhesion sites, and produce antibacterial peptides. Moreover, the gut microbiota shows immunomodulating properties through the induction of secretory IgA, maturation of dendritic cells, and regulation of Th1/Th2 balance [25]. Recent studies have also demonstrated the role of exopolysaccharides produced by *Bifidobacterium* spp. and *Lactobacillus* spp. in modulating the gut and skin epithelial immune system and in promoting the expression of various differentiation markers [64,65,66,67]. 

The distribution of specific strains belonging to the genus *Bifidobacterium* that are most prevalent throughout the human lifespan in the healthy gut of infants and adults has already been studied [68]. The knowledge about the strain-specific properties of health-promoting bacterial species belonging to human gut microbiota has allowed strategies for microbiota modulation to be defined [1,69]. 

## 3. Skin Microbiota

In the last 15 years, the advances in sampling and sequencing methods, such as whole-genome shotgun sequencing, as well as bioinformatics techniques have exponentially expanded knowledge of skin microbiomes. The skin dynamic ecosystem includes archaea; bacteria, identified thanks to next-generation sequencing technologies, including 16S ribosomal RNA gene sequencing; and fungi, identified thanks to internal transcribed-spacer sequencing technique and viruses. Bacteria are the most abundant kingdom across skin sites and specific species colonize skin niches driven by chemical conditions, but it has recently been demonstrated that different strains can exert different effects on the host [14]. Early skin colonization in infants begins at birth and recent studies have investigated whether the establishment of microbial communities may begin in utero and become specific within a few days or weeks after birth. The colonization depends on the route of delivery; babies delivered vaginally acquire microbiota from the mother’s vagina (*Lactobacillus*, *Candida albicans*) whereas babies born via caesarean section acquire microbiota from the skin (*Staphylococcus*, *Streptococcus,* and *Clostridium*). Moreover, full-term infants show a wide range of genera and differ from preterm infants (<37 weeks), who show few genera with a prevalence of skin- and gut-associated genera, such as *Staphylococcus*, *Streptococcus, Corynebacterium, Escherichia,* and *Enterococcus*. As observed for gut microbiota, during the first years of life, infants explore the external environment and are in contact with a lot of microorganisms that interact with the skin. Depending on pH, temperature, the driest or moistest zone, starts a selection process, and at the end of the process, the skin microbiota is stable. A variation in hand skin microbiota can be influenced by the frequency of handwashing and the degree of exposure to environmental elements. These external factors can influence the abundance and diversity of transient microbiota, leading to episodic exacerbations of skin disorders [15]. Thus, looking forward, the possible impact of the hygienic measures imposed to control the spread of severe acute respiratory syndrome coronavirus 2 (SARS-CoV-2) on the human microbiome is noteworthy, especially for infants’ health. On the one hand, the diversity of human microbiota can shape pulmonary viral infection progression, on the other hand, disruption in microbial sharing can be associated with dysbiosis due to the loss of bacterial diversity. However, social detachment has proven effective in controlling the pandemic and the transmission of antibiotic-resistant bacteria. Further studies should be performed to guarantee the diversity of the human microbiome while also controlling the pandemic’s spread [70].

**Figure 1:**
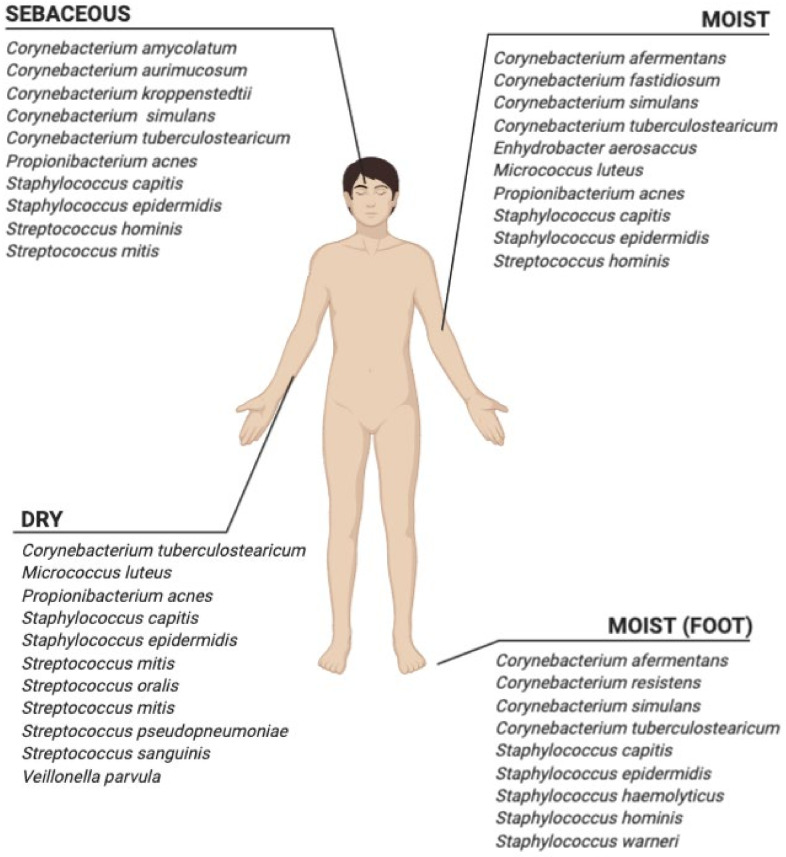
Top 10 abundant bacteria distributed according to physiological grouping of sites.

The relative abundance of genera changes during life, in particular, the genera *Corynebacterium*, *Staphylococcus*, and *Streptococcus* are predominant between 0 and 6 months; the genera *Corynebacterium*, *Cutibacterium*, *Enterococcus*, *Gemella*, *Prevotella*, *Staphylococcus*, and *Streptococcus* are predominant between 6 and 12 months; the genera *Dolosigranulum*, *Gemella*, *Granulicatella*, *Moraxella*, *Haemophilus*, *Neisseria*, *Rothia*, and *Streptococcus* are predominant between 1 and 12 years; and the genera *beta*-*Proteobacterium*, *Corynebacterium*, *Cutibacterium*, and *Flavobacterium* are predominant between 14 and 59 years [16,71]. The phyla Firmicutes (*Streptococcaceae* spp.), Bacteroidetes, and Proteobacteria (beta-Proteobacteria, gamma-Proteobacteria) are abundant in prepubescent children. The first main change in skin microbiota composition occurs during adulthood, when sexual maturation triggers physiological changes in the human skin. Sex hormones are associated with an increased activity of hormone-stimulated sebaceous glands that leads to a shift in microbiota, with a dominance of lipophilic bacteria. The skin microbiota then become stable over time (Figure 1). 

The composition of skin microbiota depends on age, site characteristics (moisture, lipid content, pH, salinity, temperature, and environmental exposure), skin pigmentation, and physiological conditions. Sebaceous skin zones (glabella, external auditory canal, manubrium and back) predominantly harbor the genera *Propionibacterium*, *Staphylococcus*, *beta-Proteobacteria*, and *Corynebacterium*; as well as *Corynebacterium* and *Flavobacteriales* dominate dry skin site zones (volar forearm, hypothenar palm). Moreover, specific species, such as *Streptococcus epidermidis*, show a tropism for human feet [72]. The adult skin microbiota is unique for each person, with some specific features in terms of genera dominance. In general, adult skin is dominated by few taxa, including genera *Staphylococcus*, *Corynebacterium*, and *Propionibacterium* [17,73].

The microbes that occupy the skin can trigger local activation of the host immune system, depending on the developmental stage of the host [14,74]. A recent study demonstrated that members of skin microbiota can regulate the expression of genes involved in epidermal differentiation and homeostasis. Individual microorganisms elicit distinct responses in the gene expression from skin tissue. Thus, for example, *Micrococcus luteus* strains are able to significantly reduce epidermal thickness [75]. In vitro and in vivo model studies demonstrated the microbiota ability to increase the production of cytokines (IL-1α and IL-1β) in a tissue model [76]. These results could be due to the ability of commensal skin microbiota to influence the behavior of cells below the epidermidis. Studies based on DNA sequencing also identified bacterial 16S rRNA in dermal and dermis adipose tissue and demonstrated that the skin microbiota extends within the dermis, where some bacteria maintain contact with cells located below the basement membrane. However, the methods used for the study did not demonstrate that live bacteria inhabit the dermis even if it is possible that live bacterial cells translocate in superficial adipose tissue. In any case, the direct bacterial–host interaction is supported by much evidence that demonstrated the ability of bacterial components (cell membrane, DNA, ATP, polysaccharides) to exert effects on host cells [77,78,79]. Replicative or non-replicative skin bacteria or produced metabolites exert a key role in the activation of toll-like receptors (TLRs) and distinct signaling pathways involved in the innate immune response [80,81,82,83].

Regulatory T cells could be involved in the regulation of early skin colonization. In neonates, early skin colonization by *Staphylococcus epidermidis* is associated with the induction of *S. epidermidis*-specific memory cells FOXP3(+) Treg, belonging to the forkhead transcription factor family, an immune response that is not observed in adults. A commensal or mutualistic behavior is shown by skin-resident microbes that play an important role in the maturation of cutaneous immunity, modulating the expression of various innate factors, such as complement components interleukin 1α (IL-1α), as well as antimicrobial peptide production (AMPs), such as cathelicidins and β-defensins by keratinocytes and sebocytes [64,74]. It is still not clear even today exactly how commensal bacteria educate the cutaneous immune system in host–bacterial interaction. Strains belonging to the species *Corynebacterium minutissimum* and *Corynebacterium tenuis* normally present in human skin are associated with erythrasma and trichomycosis axillaris. This interesting behavior could be due to the unusual features of the *Corynebacterium* spp.’s cell wall, which has an outer membrane consisting of an outer lipid bilayer of long α-branched fatty acids called mycolic acids (like those found in mycobacteria) covalently linked to the peptidoglycan meshwork. Lipomannans and lipoarabinomannans bind host glycan receptors like TLRs and C-type lectin receptors in mycobacteria, stimulating either pro- or anti-inflammatory responses [84]. The bacteria–bacterial interaction can modify the species-specific behavior of their co-residents in specific contexts. For example, *Corynebacterium striatum* is able to suppress virulence-related genes of *S. aureus*; *Corynebacterium accolens* produces a lipase able to hydrolyze triolein, inducing the release of oleic acid, which inhibits *Streptococcus pneumoniae* growth; and *Staphylococcus epidermidis* and *Staphylococcus hominis* produce antimicrobial peptides against *S. aureus*. Moreover, *Staphylococcus epidermidis* is able to activate heterologous protection, inducing keratinocytes to produce AMPs throughout the activation of strain-specific interleukin IL-17^+^CD8^+^T cells. *S. epidermidis* shows a remarkable ability to activate a selective T cell response for the non-classic major histocompatibility complex (MHC) class I, promoting homeostatic immunity that helps the innate epithelia barrier response.

It is likely that skin commensals in the gut microbiota may interact with B cells and eccrine and sebaceous glands for the production of IgA. Strains belonging to the genus *Staphylococcus* produce capsular polysaccharides, teichoic acids, and dipeptide aldehydes, which act as immune modulatory molecules [85,86].

## 4. Hypercholesterolemia and Gut Microbiota

Hypercholesterolemia is caused by dysregulation of cholesterol metabolism and represents one of the major risk factors for cardiovascular disease, which is a serious threat to human life, and the cause of 3.9 million deaths per year (45% of all causes of death) [87,88,89]. A significant positive correlation exists between hypercholesterolemia, hypertriglyceridemia, hyperuricemia, and high low-density lipoprotein (LDL). The importance of targeting serum lipids, such as triglycerides (TGs), total cholesterol (TC), high-density lipoprotein (HDL), and low-density lipoprotein (LDL), is because these parameters represent modifiable risk factors for cardiovascular diseases in humans [90,91]. The TC homeostasis is regulated through the balance between absorption, synthesis, and excretion or conversion of cholesterol into bile acids. 

In particular, the balance between primary bile acids (BA) and secondary BA is negatively correlated with plasma cholesterol concentration. Bacteria are responsible for esterification, desulfation, and oxidation, which lead to changes in hydrophobicity and BA signaling activities; bacterial hydrolases deconjugate BA, which can diffuse across the intestinal border and be captured by specific liver transporters. The bacterial 7/b-dehydrolase converts primary bile acids (cholic acid —CA— and chenodeoxycholic acid —CDCA—) into secondary BAs like lithocholic acid (LCA) and deoxycholic acid (DCA) that can be hydroxylated to form hyocholic acid (HCA) and hyodeoxycholic acid (HDCA), which decrease cholesterol absorption and the serum concentration of low-density lipoprotein cholesterol (LDL-C). The inhibition of the conversion of primary to secondary Bas, with a consequent reduction in their hydrophobicity, results in poorer reabsorption through the colonic epithelium and poorer micellization of cholesterol, with a consequent decrease in absorption. Bifidobacteria metabolize fibers and produce short-chain fatty acids, i.e., acetate, propionate lactate, and butyrate, promoting health benefits [87]. Lower plasma cholesterol levels are associated with a consumption of dietary fibers (inulin and pectin), which reduce TC and LDL-C through an increase in BA excretion and a decrease in the hepatic synthesis of cholesterol. New evidence showed that agave fructans had an impact on triglyceride synthesis in mice, while no significant differences were directly observed in TC levels. However, fructans increase the levels of SCFAs, especially acetate and also propionate and butyrate, through bifidobacterial fermentation. 

SCFAs can be used as macronutrients and act as hormone-like signals, playing a role in immunity with protective effects against cardiovascular diseases. SCFAs are absorbed from the gut and have a role in energy consumption and insulin sensitivity, especially in peripheral tissues through different G protein-coupled receptors (GPCRs). Moreover, gut microbiota can generate intermediate precursors, such as trimethylamine (TMA), which can be further metabolized by the host, generating active products that have direct effects on lipid metabolism and related disease progression [63]. 

Moreover, individual variability exists in the response to cholesterol treatment due to gene interaction and environmental effects [92]. Appropriate control of risk factors, such as lifestyle, use of functional foods, food supplements, and drugs, plays a key role in prevention. Recent findings have recognized the role of the gut microbiota in regulating host metabolism by digesting macromolecules (polysaccharides, glycosaminoglycans, glycoproteins), producing short-chain fatty acids, vitamins, and neurotransmitters [93,94], and an innate adaptative response. 

Variations in gut bacteria are associated with lipids and cholesterol blood levels in the human host because of their ability to metabolize steroids, such as cholesterol. Two periods associated with a strong modification in the amount of lipids in human hosts are birth and weaning, which are times of major change in the microbial gut composition. Epidemiological studies carried out through 16S rRNA gene or whole-genome shotgun sequencing and cross-validated by analysis on fecal taxonomy and circulating lipids have demonstrated the contribution of gut microbiome (family of *Clostridiaceae*/*Lachnospiraceae*) to variations of TC (from 1.5 to 5.6%) and LDL-C (from 0.7% to 2.8%) [89]. Anaerobic colon residents are responsible for the biosynthesis of secondary bile acids, which represents the predominant metabolic pathway for cholesterol catabolism in the human body. 

Moreover, metagenomic analysis has demonstrated that pathogenic bacteria lead to an alteration of gut barrier integrity; instead, the genera *Bifidobacterium* and *Lactobacillus* are associated with a diminution of the plasmatic cholesterol concentration. An increase in *Bifidobacteriaceae*, *Clostridiales*, and *Deferribacteriaceae* leads to a decrease in *Bacteroidetes*. Hypercholesterolemic mice supplemented with a diet of a mixture of *Bifidobacterium* and *Lactobacillus* for eight weeks showed increased propionic acid and HDL levels and decreased LDL levels compared to control mice given low-fat diets or non-supplemented high-fat diets [89,95].

## 5. Atopic Eczema/Dermatitis Syndrome and Skin Microbiota

Atopic dermatitis (AD), also known as atopic eczema, is a non-infective chronic inflammatory skin disease characterized by an itchy red rash and recurrent eczematous lesions. The term “atopy” is defined as a genetic predisposition to become sensitized and produce immune globulin E (IgE) antibodies in response to ordinary exposure to allergens. The World Allergy Organization has recently revised the nomenclature and the term “eczema” has been proposed to replace the term “atopic eczema/dermatitis syndrome”. Nowadays, the term “atopy” is only considered as the appropriate nomenclature if IgE antibody sensitization has been confirmed by blood or skin-specific tests. Eczema affects 15–20% of children during the first two years of life [73] and has shown a prevalence of 22.5% in children aged 6–7 in Ecuador, a prevalence of 24.6% in adolescents aged 14–15 in Columbia, and 2–5% of in adults in industrialized countries, with a prevalence of 10.2% in the United States.

The clinical features can be localized on the face (especially in child-adulthood), in the nappy area, and on the extensor surfaces of the knees (especially in child-adulthood), elbows, hands, and feet (especially in adulthood). A red rash, edematous papules, and vesicles, with exudate, crusting, lichenification, and hyperpigmentation or hypopigmentation are observed in infants depending on the skin type. There are different kinds of severity indices with different scores: Body Surface Area (BSA), Family Dermatology Life Quality Index (FDLQI), Eczema Area and Severity Index (EASI), and the Severity Scoring of Atopic Dermatitis (SCORAD) [41,96].

AD is characterized by an imbalance of the T-helper-2 (Th2) immune response, which causes an increase in the IgE response. In humans, a genetic deficiency of the transmembrane metalloproteinase ADAM17 is associated with eczematous dermatitis, and a mice model demonstrated that ADAM17 deficiency led to skin barrier disfunction and the development of dysbiosis, characterized by an increase in *S. aureus*, *C. bovis*, and *C. mastitis* [73]. Moreover, ADAM17 deficiency led to hyper IgE syndrome (HIES), characterized by high IgE levels in serum. These conditions led to eczematous dermatitis of the skin and pulmonary infections by *S. aureus* as well as candidiasis caused by an imbalance in the Th1 and Th17 response. 

**Figure 2:**
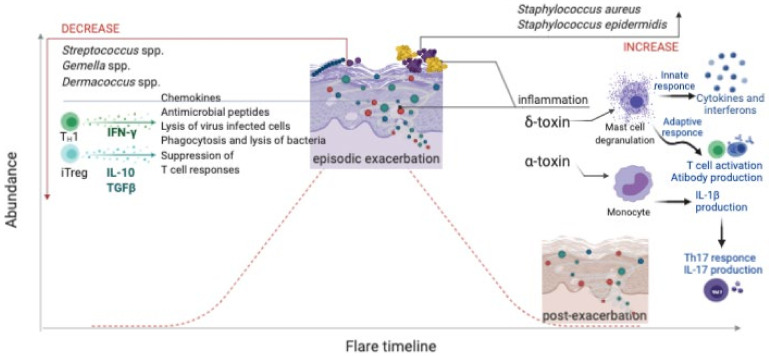
Dysbiosis in atopic dermatitis and its link to the skin immune response [71,72,73,74,75,76,77,78,79,80,81,82,83,84,85,86,87,88,89,90,91,92,93,94,95,96,97,98,99,100,101,102].

Bacterial strains of skin microbiota are able to interact with each other and can prevent colonization by pathogenic bacteria in a process called *colonization resistance*. However, some specific conditions lead to changes in the microbiota composition so that ordinarily beneficial bacteria (commensal species) become pathogenic for their hosts, a condition known as *dysbiosis*. Dysbiosis is characterized by a decreased diversity of skin commensal species, observed in many different pathological conditions, such as eczema, acne, and chronic wounds [72]. Strains belonging to the species *S. aureus* and *S. epidermidis* are commonly isolated from individuals with severe atopic dermatitis flare-ups and identified by 16S rRNA and whole-genome sequencing [97]. Studies support the notion that *Staphylococcus* spp. contribute to the initial activation of the inflammatory cascade. Whereas *Dermacoccus* spp. is completely depleted, *Gemella* spp. and *Streptococcus* spp. are reduced, and there is an overgrowth of *Corynebacterium mastitidis*, *Corynebacterium bovis*, and *S. aureus* in individuals suffering from atopic dermatitis, with a consequent increased amount of ammonia and pH levels [98]. The reduction of gammaproteobacteria leads to a decreased expression of IL-10, and a reduction of the genus *Acinetobacter* inhibits T helper 1 (Th1) and anti-inflammatory immune responses [99]. The toxins produced by *S. aureus* interact with the immune system: α-toxin induces IL-1β production from monocytes, which can promote a T helper 17 (Th17) response or cytokine IL-17 production from CD4+ T cells; δ-toxin induces mast cell degranulation (Figure 2). Murine models have demonstrated that *S. aureus* triggers adipocyte proliferation and stimulates the production of higher amounts of antimicrobial peptides, such as cathelicidin; strains belonging to this species also induce skin immune cells to produce cytokines IL-4, IL-13, and IL-22, as well as stromal lymphopoietin; these abnormal conditions seem to be responsible for alterations in the epithelial barrier [72,100,101,102,103,104,105]. 

## 6. Bifidobacteria and Their Role in Hypercholesterolemia

Among the microorganisms with claimed probiotic properties are several strains belonging to the genera *Bifidobacterium* and *Lactobacillus*. The healthy claims are strain specific and cannot be arbitrarily extended to other strains even if they belong to the same species [44,106,107]. The strain-specific claims are the results of well-designed double-blinded and placebo-controlled studies [44,106]. Moreover, the health effects can be due to produced metabolites, such as short-chain fatty acids (SCFAs) or exopolysaccharides [62,65,89,108], which can also exert different effects with respect those of the produced strain [64].

The genera *Bifidobacterium* and *Lactobacillus* are considered as very promising for the prevention of total cholesterol (TC) regulation, also in pharmaceutical approaches. A large number of in vitro and in vivo studies have shown that probiotics do have hypolipidemic effects [109]. 

*Bifidiobacterium* and *Lactobacillus* have therefore triggered great interest amongst researchers for the treatment of cardiovascular disease. Animal models have demonstrated that these strains are able to reduce hepatic cholesterol levels, improve the adsorption of nutrients, and increase the production of short-chain fatty acids (SCFAs) [89]. SCFAs are produced mainly by *Bifidobacterium*, *Bacteroides*, *Clostriudium*, *Provotella*, and *Ruminococcus* and are implicated in sympathetic system activation and the maintenance of lymphocyte influx to the gut epithelium. A reduction in acetate levels is involved in hypertension in sleep apnea. Propionate administration leads to a reduction of systolic and diastolic blood pressure. Instead, butyrate administration ameliorates vascular function. Moreover, SCFAs can activate G-protein coupled receptors (Gpr41, Gpr43, Gpr109a, and Olfr78), regulating renin secretion and blood pressure [62,108]. In vivo studies have demonstrated that the supplementation of acetate, butyrate, and propionate in rats/Syrian hamsters administered high levels of dietary cholesterol led to a decrease in CT levels and the LDL-C/HDL-C ratio and an increase in the fecal excretion of Bas, such as CA, CDCA, LCA, and DCA [89]. 

Moreover, they can modulate the peripheral expression of the *bdnf* gene in zebrafish [110]. A rat model demonstrated that *Bifidobacterium longum* BB536 significantly reduced total cholesterol, low-density lipoprotein cholesterol, high-density lipoprotein cholesterol, very low-density lipoprotein cholesterol, and atherosclerotic index levels as well as liver lipid deposition, adipocyte size, and positively affected liver and kidney function [111].

In 2009, an Italian research group studied the activity of *Lactobacillus plantarum* and *Lactobacillus paracasei*, isolated from Castelmagno D.O.P. cheese, in lowering cholesterol in vitro (without presence of bile) [26]. Another study confirmed the cholesterol-lowering effects of *L. plantarum* studied in rats [30]. In 1985, Gilliland et al. noted that cholesterol could be assimilated by *Lactobacillus acidophilus*. The study demonstrated that *Lactobacillus acidophilus* RP 32 significantly inhibited the increase of serum cholesterol in pigs, and the researchers hypothesized a direct action of the microorganism on the molecule [31]. Another in vitro study in 2000 confirmed this hypothesis [27]. In 2018, an article [112] was published that highlighted a decrease in circulating cholesterol in a murine model treated with *Lactobacillus rhamnosus* BFE5264 isolated from Maasai fermented milk. This discovery could at least partly explain the results of a 1975 group-control study in which Mann et al. (treatment group fed with typical Maasai fermented milk with additional fat and a second control group with milk and placebo) noted that there was no difference in the total cholesterol of each of the groups, assuming an intrinsic factor in milk helps the body to break down exogenous cholesterol [113].

However, there is some controversy in human clinical studies about the lipid-lowering effect of probiotics. Some research has argued against this role [109]. A recent metanalysis by Wang et al. (2018) compared 32 studies with 1971 participants and showed that there is a decrease in total cholesterol when lactobacilli and bifidobacteria are used as dietary supplements in various foods. This overall reduction in the sum of all studies was found to be on average −13.27 mg/dL, 95% CI (−16.74; −9.80), *p* < 0.05 [109]. Further studies should be performed to evaluate how bifidobacterial and SCFA production can modulate TC levels and vascular disease progression. Table 1 shows the main clinical and experimental studies on atopic dermatitis and hypercholesterolemia recently performed on bifidobacteria alone and in combination with lactobacilli.

## 7. Bifidobacteria and Their Therapeutic Potential in Eczema

The genus *Bifidobacterium* has been shown to be reduced in the gut microbiota of infants, children, and young adults with eczema. Skin disorders are related to a defect in the barrier beyond the skin and the intestinal mucosa, where gut microbiota contribute to the maintenance of intestinal permeability. On the contrary, the gut barrier breakdown leads to an intestinal permeability increase and a consequent bacterial and endotoxin (lipopolysaccharide, LPS) translocation into the systemic circulation, leading to immune disorders [114]. A reduction of plasmatic LPS levels was observed in patients treated with *Bifidobacterium breve* BR03 in combination with *Lactobacillus salivarius* LS01 [115]. Evidence indicates that strains belonging to the genus *Bifibobacterium* and *Lactobacillus* have immunomodulatory effects, stimulating T helper 1 (Th1) cytokines and suppressing the T helper 2 (Th2) response, which is increased in chronic eczema. In particular, the production of IL-10 and TGF-beta (tumor growth factor) is stimulated [41]. Moreover, strains belonging to the species *Bifidobacterium longum* are able to promote the production of antimicrobial peptides in keratinocytes and to produce metabolites, such as lipids, which can help skin commensals avoid pathogenic strains’ colonization [17]. The development of allergic diseases in children seems to be related to a defect in the early stimulation of Th1 cells. Eczema is considered a multifactorial disease, associated with epigenetic, genetic, developmental, and environmental factors. Studies have demonstrated that infants suffering from eczema show less gut colonization by strains belonging to the genus *Bifidobacterium* and *Lactobacillus*, with a prevalence in skin microbiota of strains belonging to *Clostridium* spp. and *Staphylococcus* spp. as compared to healthy children [96,115,116]. Strains belonging to the genus *Bifidobacterium* and *Lactobacillus* are able to interact with the immune system, modulating Th1 cytokine production and the Th2 response. They also show an ability to regulate regulatory T cells (Tregs), diminishing the inflammatory pathway; in particular, they are able to decrease immune globulin E (IgE), interferon-gamma, and eosinophilia [117,118,119,120]. *Bifidobacteria adolescentis* treatments reduced ear and skin thickness and suppressed eosinophils and mast cell infiltration. Th1- and Th2-type responses were regulated and the Tregs population was promoted in the spleen by *B. adolescentis* treatments. The strains *Bifidobacterium adolescentis* Ad1, *B. adolescentis* Ad2, *B. adolescentis* Ad3, *B. adolescentis* Ad4, *B. adolescentis* Ad5, and *B. adolescentis* Ad6 led to an increase in the relative abundance of the genus *Lactobacillus* and to a decrease in the relative abundance of the genera *Dorea* and *Pediococcus*. Moreover, the strains *B. adolescentis* Ad1, *B. adolescentis* Ad3, and *B. adolescentis* Ad6 decreased ear thickness; and the strains *B. adolescentis* Ad1 and *B. adolescentis* Ad3 alleviated AD clinical symptoms. The strains *B. adolescentis* Ad1, *B. adolescentis* Ad4, *B. adolescentis* Ad5, and *B. adolescentis* Ad6 significantly reduced serum immune globulin E (IgE) levels and IL-4 levels [121].

Lise et al. (2019) [96] reported a clinical case regarding a female 18-month-old child who showed xerosis, Dennie–Morgan double fold, and areas of erythema and lichenification in the antecubital fossae, abdomen, and legs. The scoring atopic dermatitis index (SCORAD) was 60.15, body surface area (BSA) was 60%, family dermatology life quality index (FDLQI) was 18, and IgE was 140 kU/L (normal value: <60 kU/L). The young patient was treated with a mixture of *Bifidobacterium lactis* HN019, *Lactobacillus acidophilus* NCFM, *Lactobacillus rhamnosus* HN001, and *Lactobacillus paracasei* LPC37 once a day after a bath for two weeks. After treatment, all parameters improved: the SCORAD was 4.95 and BSA 0% while the FDLQI was 8.

Moreover, it has been demonstrated that non-replicative *Bifidobacterium longum* NCC2705 and *Bifidobacterium longum* NCC3001 (heat treated), *Bifidobacterium longum* BL/81, and sonicated *Bifidobacterium longum* BL/84 cells promote differentiation markers (keratin KRT1, KRT10, and transglutaminase TGM1) of human keratinocytes (NHEKs) during pre-confluent and confluent stadiums. Moreover, the expression of antimicrobial peptides, such as beta-defensin-1 and BDEF, as well as the molecules involved in wound healing, such as cathepsins B, D, and H, was significantly elevated in post-confluent NHEKs after treatment with *Bifidobacterium longum* non-replicative strains and extracts. These results were confirmed by the levels of mRNA transcripts. The mechanism could be related to the ability to increase intracellular calcium levels, inducing a calcium influx from the extracellular medium [67]. One other mechanism proposed in recent literature is an increased expression of brain-derived neurotrophic factor (BDNF) starting from butyrate, which is one of the main SCFAs derived from oligosaccharides, such as fructooligosaccharides (FOSs) and galactolygosaccharides (GOSs), through metabolism by bifidobacteria [122,123,124,125]. Thus, for example, *Bifidobacterium longum* BL986 orally administered in mice led to an increase in the mRNA expression of BDNF and caused a decrease in glutathione-disulfide reductase (*Gsr*), superoxide dismutase (*Sod*), catalase (*Cat*), tumour necrosis factor (*Tnf*), *Il-6*, and *Il-1b* expression in peripheral tissue [126]. The suppression of *Il-1b* due to *Bifidobacterium breve* B-3 metabolic activity increased cathepsin L-like protease activity, reducing the incidence of atopic eczema [104,127,128,129]. Future studies are needed to further evaluate how live or killed bifidobacteria can be used in topic formulations to ameliorate the progression of atopic dermatitis symptoms thanks to a restored balance between friend and foe strains.

**Table 1:** Main clinical and experimental studies and principal strain-specific effects of bifidobacteria, used alone and in combination with lactobacilli, on hypercholesterolemia and atopic dermatitis.

Strain	Dosage/Dye	Study Design/Subjects	Principal Results	References
*Bifidobacterium animalis* subsp. lactis BB-12	1 × 10^9^ CFU/g ^1^	Infants	↓ Neutral lipids in plasma ↑ Phospholipids	Kankaanpaa et al. (2002) [130]
*Bifidobacterium longum* BB536	3 × 10^7^ CFU/mL ^3^	Rat model	↓ Total cholesterol ↓ Liver lipids deposition ↓ Adipocyte size	Al-Sheraji et al. (2015) [111]
*Bifidobacterium infantis* 35624	1 × 10^10^ CFU/mL ^3^	Human	↓ C-reactive protein level ↓ TNF-alfa	Groeger et al. (2013) [131]
*Bifidobacterium breve B-3*	2 × 10^9^ CFU/mouse ^2^	Mouse model	↓ IL-1beta ↓ Claudin-1 ↓ skin damage ↑ TJs integrity	Satoh et al. (2015) [128]
*Bifidobacterium adolescentis* Ad1-6	1 × 10^9^ CFU/0.2 mL	Mouse model ^4^	↓ IgE ↓ IL-4 ↓ AD-simptoms	Fang et al. (2019) [131]
*Bifidobacterium breve* BR03 *Lactobacillus salivarius* LS01	1x10^9^ CFU/g ^5^	Human	↓ SCORAD	Iemoli et al. (2012) [132]
*Bifidobacterium longum* CECT 7347 *Bifidobacterium lactis* CECT 8145 *Lactobacillus casei* CECT 9104	1 × 10^9^ CFU/mL ^6^	Human ^7^	↓ AD SCORAD	Navarro-López et al. (2018) [133]
*Bifidobacterium breve* Bbi99 *Lactobacillus rhamnosus* GG *Lactobacillus rhamnosus* LC705 *Propionibacterium freudenreichii spp. shermanii* JD	2 × 10^8^ CFU/mL 5 × 10^9^ CFU/mL 5 × 10^9^ CFU/mL 2 × 10^9^ CFU/mL ^8^	Infants	↓ AD rates	Kukkonen et al. (2007) [127]
*Bifidobacterium animalis* subspecies *lactis* CUL34 *Bifidobacterium bifidum* CUL20 *Lactobacillus salivarius* CUL61 *Lactobacillus paracasei* CUL08	1 × 10^10^ CFU/mL ^9^	Infants	↑ AD prevention	Allen et al. 2014 [134]
*Bifidobacterium lactis* HN019 *Lactobacillus acidophilus* NCFM *Lactobacillus rhamnosus* HN001 *Lactobacillus paracasei* LPC37	1 × 10^10^ CFU/mL ^9^	Infants ^10^	↓ SCORAD ↓ BSA ↓ FDLQI	Lise et al. (1992) [96]
*Bifidobacterium breve* Bbi99 *Lactobacillus rhamnosus* GG *Lactobacillus rhamnosus* LC705 *Propionibacterium freudenreichii spp. shermanii* JD	2 × 10^8^ CFU/mL 5 × 10^9^ CFU/mL 5 × 10^9^ CFU/mL 2 × 10^10^ CFU/mL ^8^	Children ^11^	↑ AD prevention	Kuitunen et al. (2009) [135]
*Bifidobacterium animalis* subsp. lactis BB-12 *Lactobacillus rhamnosus* GG	1 × 10^10^ CFU/mL	Human adults ^12^	↑ AD prevention	Rautava et al. 2006 [136]
*Bifidobacterium longum* BL999 *Lactobacillus rhamnosus* LPR	1 × 10^10^ CFU/mL	Human adults ^12^	↑ AD prevention	Rautava et al. 2012 [137]
*Bifidobacterium lactis BB-13* *Lactobacillus rhamnosus* GG	1 × 10^10^ CFU/mL	Human adults ^12^	↑ AD prevention	Huurre et al. (2008) [138]
*Bifidobacterium lactis* *Lactobacillus rhamnosus*	2 × 10^10^ CFU/g ^6^	Children ^13^	↓ SCORAD	Sistek et al. (2006) [139]
*Bifidobacterium breve* YIT 12272 *Lactococcus lactis* YIT 2027 *Streptococcus thermophilus* YIT 2021	5–6 × 10^10^ CFU/100 mL ^14^	Human adults ^12^	↑ Cathepsin L-like activity ↑ Hydration level ↓ Serum phenol ↓ Urine phenol	Kano et al. (2013) [129]

^1^ Administration: 73 mL/kg daily for 7.3 months; ^2^ administration: once a day for 7 weeks; ^3^ administration: daily for 8 weeks; ^4^ six-week-old, female, and specific pathogen-free grade C57bl/6 mice; ^5^ ratio (1:2) twice a day for 12 weeks; ^6^ ratio (1:1:1) administered for 12 weeks; ^7^ topical treatment; ^8^ administration: twice daily 2–4 weeks; ^9^ total amount administered daily for two weeks; ^10^ female infants aged 18 months; ^11^ <5 years Double-lind randomized placebo controlled; ^12^ Double-blind randomized placebo controlled; ^13^ 1–10 years of age; ^14^ administered daily for 4 weeks.

## 8. Conclusions

Bifidobacteria play a central role in human physiology due to their metabolic capability, especially that related to SCFA production. SCFAs, mainly produced by *Bifidobacterium* spp., contribute to cholesterol reduction and have trophic properties thanks to butyrate’s ability to increase prodifferentiation and proangiogenic factors [63]. Moreover, in vitro studies have demonstrated that the metabolic products of strains used as a probiotic component in food supplements, for example, the Niemann-Pick C1 Like 1 (NPC1L1) protein, are able to downregulate bile salt deconjugation. The usefulness of strains belonging to the genus *Bifidobacterium* and *Lactobacillus* in reducing serum cholesterol appears, as explained above, to have been confirmed by several studies and clinical trials. Moreover, when used in combination, the role of lactobacilli in the improved bifidobacterial survivor should be clarified by investigating the function of hydrolases in bile acid deconjugation. The mechanisms that make this happen still need further investigation.

The prodifferentiation and proangiogenic ability of butyrate to promote epithelial cells differentiation, as well as the capability of exopolysaccharides to modulate the gut and skin epithelial immune system and to promote the expression of various epithelial differentiation markers, suggests new promising strategies to prevent or treat eczema and disorders like dermatitis and hypercholesterolemia [51,52,53,54,55,56,57,58,59,60,61,62,63]. However, for the sake of completeness, it must also be emphasized that there are some case reports in the literature of septicemia in patients due to probiotic dietary supplements. These were severely immunocompromised patients with intestinal inflammatory damage [140]. These case reports thus suggest caution in the use of living bacteria as supplements in frail and immunocompromised patients.

Future work may focus on investigations of other cholesterol-lowering properties, including screening for bile salt hydrolase activity. In terms of cholesterol assimilation, the specific mechanism by which cholesterol is removed from the supernatant should be determined. Ideally, a probiotic should be developed that would affect multiple targets, using bile salt hydrolase activity, reducing 3-hydroxy-3-methylglutaryl-coenzyme A (HMG-CoA) reductase activity, and assimilating cholesterol. Moreover, a probiotic formulation could be developed as a combination therapy with pharmaceuticals, such as statins.
